# The predictive value of fibrinogen in the occurrence of mild cognitive impairment events in patients with diabetic peripheral neuropathy

**DOI:** 10.1186/s12902-022-01185-2

**Published:** 2022-11-02

**Authors:** Yong Zhuang, Huibin Huang, Zhenfei Fu, Jinying Zhang, Qingyan Cai

**Affiliations:** 1 Department of Endocrinology, The Second Affiliated Hospital of Fujian Medical University, No.950 Donghai Street, Fengze District, Quanzhou City, Fujian Province China; 2 Department of Neurology, The Second Affiliated Hospital of Fujian Medical University, Quanzhou, 362000 China

**Keywords:** Diabetic peripheral neuropathy, Mild cognitive impairment events, Fibrinogen, Predictive value

## Abstract

**Background:**

Research suggests that fibrinogen (Fib) is related to mild cognitive impairment (MCI) and diabetic peripheral neuropathy (DPN) and the risk of MCI in patients with DPN is greatly increased, although no studies have evaluated the predictive value of Fib for the risk of MCI in patients with DPN.

**Methods:**

This prospective observational clinical study enrolled 207 type 2 diabetes mellitus (T2DM) patients, who were divided into diabetes with no neuropathy (102 cases) and diabetes with neuropathy (105 cases) groups. Meanwhile, 90 healthy unrelated subjects were recruited as controls. The incidence of MCI in the DPN patients was followed up for 2 years. Divide patients in the DPN group into subgroups according to whether MCI occur, use multivariate logistic regression to analyze independent factors of MCIs in DPN patients within 2 years, and use ROC curve to analyze the predictive value of Fib for MCI in DPN patients.

**Results:**

Fib levels were significantly higher in diabetic subjects with neuropathy compared with those without (*P* < 0.001). In further subgroup analysis of DPN patients who were divided according to the occurrence of MCI, baseline data of the MCI subgroup showed Fib levels were higher than that in the non-MCI group while education levels declined (*P* < 0.001). The education level and increased Fib levels were independent factors for the occurrence of MCI within 2 years after the onset of DPN (*OR* = 0.769, 95% *CI*: 0.605 ~ 0.968, *P* = 0.037; *OR* = 2.674, 95% *CI*: 1.094 ~ 3.168, *P* = 0.002). The ROC curve indicated that the predictive value of Fib was (*AUC* = 0.764, 95% *CI*: 0.671 ~ 0.842, *P* < 0.001).

**Conclusions:**

Fib may function as a predictor for assessing the risk of MCI in DPN patients.

## Introduction

It has been acknowledged that diabetics mellitus is the fifth leading cause of death in the world, while diabetic peripheral neuropathy (DPN) is a common chronic complication of diabetes, with an incidence of 60% to 90% [1–3]. Cognitive impairment is considered to be a complication of central nervous system damage in diabetes mellitus. DPN and cognitive impairment in type 2 diabetes mellitus (T2DM) may share similar pathophysiologic changes, such as neurovascular mechanisms, insulin signaling and hyperglycemia and so on [4, 5]. DPN may be an independent risk factor for cognitive impairment in T2DM patients. Patients with DPN show more severe cognitive impairment, especially in executive functions, concentration and attention [6, 7].

Fibrinogen (Fib) is a plasma protein which also reflects the inflammatory state of the body and plays an important role in the inflammatory response. Fib has been of interest as not only a marker of vascular pathology but also as an active contributor to neurodegenerative diseases [8, 9]. In addition, DPN is known to be associated with oxidative stress, immune, metabolic abnormalities and inflammatory responses [10]. We have confirmed that Fib and DPN are closely related [11]. The correlation between DPN and Fib has also been confirmed in several studies [12–14]. Cognitive impairment is closely connected to neurodegeneration, vascular pathology and neuroinflammation. Fib is also strongly linked to cognitive impairment [15]. Studies [16, 17] indicated that high levels of Fib are linked to Alzheimer's disease, brain atrophy and cognitive deficits, whereas Hainsworth AH et al. [18] discovered that extravascular Fib is toxic to cognitive function in individuals with histological lesions. In animal experiments, abnormal deposition and persistence of Fib may lead to amyloid-β deposition, decreased cerebral blood flow, increased neuroinflammation, and ultimately neurodegeneration [19].

However, mild cognitive impairment (MCI) is an early state of cognitive impairment and dementia [20]. MCI is often overlooked because of its insidious condition. Early identification and treatment of MCI can significantly reduce the incidence of dementia and improve long-term prognosis. Therefore, it is of great significance to study the related serological markers of DPN and MCI. In addition, little data is available for Chinese individuals who face an increasing incidence of diabetes [21]. Therefore, we evaluated the predictive value of Fib for the risk of MCI in patients with DPN.

## Materials and methods

### General information

A total of 207 subjects who met the 1999 World Health Organization (WHO) type 2 diabetes diagnostic criteria and were registered consecutively as inpatients or outpatients with our hospital between March 2018 and March 2019 were randomly enrolled in this study [11]. And 90 healthy subjects were randomly included as a control group. Randomization was performed by a person with no other role in the study who randomly drew a sealed opaque envelope out of a container to determine participant allocation. All participants were informed and signed the consent form. This study was approved by the ethics committees of the university and the hospital. The exclusion criteria were age < 30 years or > 70 years, illiteracy, inflammatory lesions of the central nervous system, mental retardation, lactation or pregnancy, trauma surgery, peripheral vascular disease, trauma, acute infection, diabetic ketosis, severe liver or kidney damage, tumour, long-term alcohol abuse, vitamin B12 deficiency, blood disease and osteoarthritis. No history of diabetes, glycated haemoglobin < 5.6% and fasting blood glucose < 5.6 mmol/L were the inclusion criteria for the normal control group of this study.

### Method

#### Clinical feature measurement

The study tested each participant according to standard procedures by the same experienced physician. All inspections and tests are carried out in a quiet and comfortable laboratory.

All patients underwent a physical examination and had a complete history of neurological symptoms. All participants were examined using electromyography (EMG) instrument (Keypoint 9033A07, Denmark) [11].

Each enrolled participant was screened by a neurologist according to the diagnostic criteria for MCI proposed by Petersen [22], and the diagnosis of MCI is mainly based on clinical symptoms and scale screening. The criteria are (1) memory complaint, preferably corroborated by an informant, (2) objective memory impairment for age, (3) relatively preserved general cognition for age, (4) essentially intact activities of daily living, and (5) not demented. Meanwhile, combined with Montreal Cognitive Assessment (MoCA) scale scores.

Standing height and weight were measured on the same all-in-one scale without shoes on before breakfast. Calculate BMI value by weight (kg)/ height (m)^2^. After a 15-min rest, the blood pressure of each seated subject's right arm was measured with a mercury sphygmomanometer.

All participants stopped using antiplatelet and anticoagulant drugs 2 weeks ago, and collected venous blood from the antecubital vein in the morning after 10–12 h of fasting. Fib was collected and measured using a blood coagulation meter (FAC21A-UW; Ltd, Taiwan) according to the instruction of the manufacturer. Blood lipids, fasting plasma glucose, serum creatinine, and liver and kidney function were tested by an automatic biochemical analyser (Cobas 8000; Roche, Germany). Serum vitamin B12 was determined using an automated assay (Maglumi 4000; China). HbA1c was assessed using high-performance liquid chromatography (D10; Bio–Rad, Berkeley, CA). The urinary albumin concentration was measured using immunonephelometry (DCA2000; Bayer, Leverkusen, North Rhine-Westphalia, Germany). The urinary creatinine and albumin was measured using the alkaline picrate method. Obtain urinary albumin-creatinine ratio (UACR) by calculating albumin (mg)/creatinine (g). Estimated glomerular filtration rate is based on the Cockcroft equation to calculate endogenous creatine clearance (Ccr): Ccr = {[140– age (years) × body weight (kg)]/[0.818 × serum creatinine (Scr, µmol/L)]} for males and × 0.85 for females.

#### Followed up for 2 years

The enrolled participants were followed up for 2 years in the form of outpatient follow-up or readmission. The neurologist reassessed, when the participants met the diagnostic criteria for MCI proposed by Petersen [22] and 19 points ≤ MoCA < 26 points were judged as MCI occurrence. The participants were divided into two subgroups according to the occurrence of MCIs, namely the MCI group and the non-MCI group.

### Statistical analysis

We used SPSS version 19 (SPSS Inc., IBM, Chicago) for statistical analysis. The data is expressed as the mean (SD) for normally distributed data. The count data were compared using the chi-square test. Multiple comparisons among groups were assessed using one-way analysis and comparisons between two groups (LSD method) for variables. A t test was used to compare the differences between the two groups. Fib was added to the logistic regression model to control for possible confounders. Receiver operating characteristic (ROC) analysis was performed using MedCalc Software version 19.04 (MedCalc Software bvba, Ostend, Belgium) to assess the predictive value of Fib for the risk of MCI in patients with DPN. The optimal cutoff point for Fib was determined by calculating the area under the curve (AUC). *P* < 0.05 was considered statistical significance.

## Results

The study was completed by 297 subjects, all of whom were followed up, which included 90 healthy control subjects, 102 diabetes without neuropathy, and 105 diabetes with neuropathy (Table 1). Among all subjects, there were no differences between the three groups in the following variables: age, BMI, blood pressure (DBP and SBP), sex ratio, blood lipids [low-density lipoprotein (LDL) cholesterol, high-density lipoprotein (HDL) cholesterol and total cholesterol (TC)], kidney and liver function [UACR and Ccr, aspartate transaminase (AST), alanine transaminase (ALT)], education level and vit B12 (Table 1). A comparison of the groups revealed that the Fib was the highest in the DPN group (Table 1). The disease course was higher in the DPN group than in other groups. Incidence of smoking history and HbA1c were also higher in the DPN group than in other groups (Table 1).

**Table 1:** Comparison of clinical features between different groups

Group	Healthy control	*P* value^1^	Diabetes without neuropathy	*P* value^2^	Diabetes with neuropathy	*P* value^3^
Case (male/female)	48/42	0.723	57/45	0.791	54/51	0.521
Age (years)	51.3 ± 8.0	0.749	51.0 ± 9.4	0.750	51.7 ± 7.3	0.509
Disease course (years)	—	—	5.5 ± 3.2	—	7.9 ± 3.8	0.000
Smoking (N/Y)	59/31	0.871	68/34	0.024	52/53	0.012
Education level (years)	9.9 ± 2.9	0.775	9.7 ± 2.9	0.401	9.5 ± 3.0	0.568
SBP (mmHg)	122 ± 7	0.091	124 ± 9	0.365	123 ± 10	0.410
DBP (mmHg)	70 ± 6	0.910	70 ± 8	0.988	70 ± 6	0.894
BMI (kg/m ^2^)	24.4 ± 1.5	0.490	24.5 ± 1.5	0.670	24.5 ± 1.4	0.781
FPG (mmol/L)	4.7 ± 0.5	0.000	8.2 ± 1.3	0.000	8.2 ± 1.5	0.928
HbA1c (%)	4.8 ± 0.4	0.000	7.7 ± 1.2	0.000	8.8 ± 1.9	0.000
TC (mmol/L)	4.8 ± 0.6	0.297	4.9 ± 0.6	0.196	4.8 ± 0.8	0.800
LDL-C (mmol/L)	2.6 ± 0.7	0.430	2.7 ± 0.7	0.616	2.7 ± 0.9	0.762
HDL-C (mmol/L)	1.3 ± 0.3	0.243	1.3 ± 0.4	0.285	1.3 ± 0.4	0.913
ALT (IU/L)	24 ± 4	0.831	24 ± 3	0.458	24 ± 2	0.586
AST (IU/L)	22 ± 4	0.892	22 ± 3	0.346	21 ± 4	0.265
Vit B12 (pmol/L)	531 ± 169	0.933	534 ± 196	0.590	546 ± 206	0.638
UACR (mg/g)	21.4 ± 2.8	0.594	21.6 ± 3.3	0.962	21.4 ± 2.8	0.613
eGFR [ml/(min·1.73m^2^)]	126.8 ± 38.5	0.387	122.5 ± 27.3	0.277	121.4 ± 36.5	0.823
Fib(g/L)	2.98 ± 0.58	0.400	3.06 ± 0.51	0.000	4.38 ± 0.83	0.000

^1^*P*, healthy control vs. diabetes without neuropathy. ^2^*P*, healthy control vs. diabetes with neuropathy. ^3^*P*, diabetes without neuropathy vs. diabetes with neuropathy

A total of 34 MCI events occurred in the diabetes with neuropathy group after 2 years of follow-up (no MCIs occurred in either the healthy control group or diabetes without neuropathy group). Divide patients in the DPN group into subgroups according to whether MCI occurred, baseline data of the MCI subgroup showed higher levels of Fib compared with patients without while education levels were lower (*P* < 0.001; *P* = 0.008) (Table 2).

**Table 2:** Comparison of baseline characteristics of MCIs and non-MCIs between two subgroups

Subgroup	MCI	non-MCI	*P* value
Case (male/female)	15/19	39/32	0.300
Age (years)	53.0 ± 6.3	51.1 ± 7.7	0.218
Disease course (years)	8.6 ± 4.1	7.6 ± 3.6	0.198
Smoking (N/Y)	16/18	36/35	0.727
Education level (years)	8.4 ± 2.6	10.0 ± 3.0	0.008
SBP (mmHg)	124 ± 11	122 ± 10	0.332
DBP (mmHg)	70 ± 6	69 ± 6	0.668
BMI (kg/m ^2^)	24.4 ± 1.3	24.5 ± 1.4	0.720
FPG (mmol/L)	8.1 ± 1.3	8.3 ± 1.6	0.615
HbA1c (%)	9.1 ± 2.6	8.7 ± 1.6	0.326
TC (mmol/L)	4.7 ± 1.1	4.9 ± 0.5	0.171
LDL-C (mmol/L)	2.5 ± 1.0	2.8 ± 0.8	0.205
HDL-C (mmol/L)	1.2 ± 0.4	1.3 ± 0.3	0.343
ALT (IU/L)	24 ± 2	24 ± 3	0.367
AST (IU/L)	21 ± 3	21 ± 5	0.531
Vit B12 (pmol/L)	543 ± 202	548 ± 209	0.914
UACR (mg/g)	21.2 ± 3.0	21.5 ± 2.7	0.643
eGFR [ml/(min·1.73m^2^)]	124.8 ± 44.9	119.8 ± 31.9	0.507
Fib(g/L)	4.81 ± 0.84	4.18 ± 0.74	0.000

The Fib was further assessed in relation to MCI in a multivariate model, controlling for education level and other variables that may potentially influence the Fib level or MCI, including the disease course, age, education level, smoking, HbA1c, LDL and vit B12. After adjustment, the Fib was still independently associated with MCI (odds ratio 2.674 [1.094 ~ 3.168], *P* = 0.002) (Table 3). Correspondingly, the education level was also independently associated with MCI (odds ratio 0.769 [0.605 ~ 0.968], *P* = 0.037) (Table 3).

**Table 3:** Multivariate logistic regression of MCIs in DPN patients

Covariables	*OR*	*95% CI*	*P* value
Disease course	1.030	0.902 ~ 1.155	0.653
Age	0.980	0.901 ~ 1.061	0.631
Education level	0.769	0.605 ~ 0.968	0.037
Smoking	1.294	0.452 ~ 2.823	0.597
HbA1c	1.158	0.913 ~ 1.460	0.239
LDL	0.620	0.368 ~ 1.071	0.089
Vit B12	1.000	0.998 ~ 1.003	0.804
Fib	2.674	1.094 ~ 3.168	0.002

**Fig. 1:**
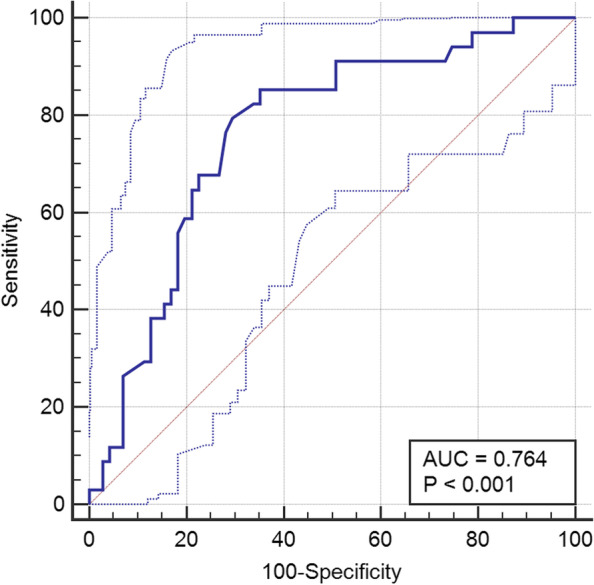
ROC curve of Fib

An ROC curve was used to derive a cutoff point above which the Fib can be predicted the occurrence of MCI in DPN patients as illustrated (Fig. 1). The ROC curve for determining the cutoff value for whether or not MCI will occur yielded an area under the curve of 0.764 (95% confidence interval, 0.671 ~ 0.842, *P* < 0.001) with a standard error of 0.049. The optimal cut-off point was 4.12 g/L for the Fib, with a sensitivity of 85.29% and a specificity of 64.79%.

## Discussion

Based on a cohort of Chinese DPN patients, this prospective study confirmed that Fib level was associated with DPN and the incidence of MCI in DPN patients was higher than that in DM patients and the general population. Furthermore, in this study, the baseline data of Fib level which DPN patients with MCI was significantly higher than that without. More importantly, the increased Fib level was an independent factor for the occurrence of MCI in DPN patients during follow-up.

The size of the diabetics mellitus population has been dramatically increasing worldwide. As a common complication of diabetics mellitus, DPN has a huge base and the incidence rate reported in China is as high as 85%. MCI is an intermediate state [23, 24] between normal aging and dementia which can evolve to dementia, mostly in the form of Alzheimer’s disease [25, 26]. Dementia leads to a marked decrease in quality of life and consumes a lot of medical resources. If this stage can be identified early and actionable interventions to delay or prevent its onset can be developed, the progress of MCI to dementia could be alleviated. Therefore, more trials are needed to discover some strategies for the prevention and delay of MCI. Both DPN and MCI are diabetic complications. Studies found that DPN and MCI share similar pathophysiologic changes such as the adverse effects of advanced glycation end products on body metabolism include oxidative stress, impaired cell function, plaque modification, etc. [27, 28]. And the risk of MCI in patients with DPN is greatly increased. Therefore, understanding the occurrence of MCI in patients with DPN is one of the important issues.

Fib is a plasma glycoprotein synthesized which is not only a substrate for thrombin to participate in the coagulation process, but also reflects the inflammatory state of the body and plays an important role in the inflammatory response [13, 29]. Elevated levels of Fib indicates the increase of coagulation viscosity and the existence of inflammatory reaction. The pathogenesis of DPN remains unclear among which microvascular changes and metabolic pathway abnormalities are considered to play an important role in the occurrence and development of DPN [30, 31]. Increased secretion of plasminogen activator inhibitors under the stimulation of insulin and blood sugar, and increased secretion of plasminogen activator inhibitors due to damage to the vascular endothelium, these lead to a hypercoagulable state and an inflammatory response that ultimately promotes the occurrence and development of DPN [32]. Our previous study has confirmed that Fib was closely related to DPN [11]. The result of this study further demonstratedt that Fib was related to DPN. In this study, patients with DPN had poorer blood sugar control than those without, and had a higher proportion of smoking.

On the other hand, high Fib level is associated with cognitive decline and dementia which may be caused by factors such as white matter lesions, silent infarction, leukoaraiosis and cerebral hypoperfusion [33–35]. In Alzheimer's disease, Fib deposits in the brain parenchyma and cerebral vessels which may promote and contribute to neuroinflammation [19, 36]. Bordignon [37] found that high Fib levels may predict the risk of cerebrovascular events in older people with cognitive impairment. Our study further compared the baseline data on whether DPN patients developed MCI through 2-year follow-up. In the absence of differences in blood glucose, age, and smoking history, the results showed that DPN patients who developed MCI had significantly higher Fib level than those who did not. Moreover, this study found that both Fib level and education level were independent factors for the occurrence of MCI in DPN patients, suggesting that high levels of Fib could be a potential predictor of MCI in DPN patients. The AUC of Fib for predicting the occurrence of MCI in DPN patients was 0.764, which more than the 0.7 suggested that Fib has a certain predictive value as an assessment of the risk of MCI in DPN patients. The cut-off value of Fib was 4.12 g/L (greater than 4.12 g/L), suggesting that when Fib level is greater than 4.12 g/L (reference range: 2.0–4.0 g/L), it may play a more important role in the occurrence of MCI in DPN patients.

Our study suggests that Fib plays an important role in the development of MCI in DPN patients, from a clinical point of view, strengthens the need for a personalized approach when dealing with DPN patients, even in the risk. Clinicians can monitor Fib and make timely adjustments to prevent the occurrence of MCI in DPN patients based on individualized circumstances. Of course, whether reducing Fib level can reduce the risk of MCI events in DPN patients requires further intervention studies.

## Conclusions

In conclusion, Fib is not only associated with DPN and MCI, but more importantly, Fib is an independent risk factor for the occurrence of MCI in DPN patients, and when Fib is greater than 4.12 g/L, it may play a greater role in the occurrence of MCI. It holds promise as a predictor for assessing the risk of MCI events in DPN patients. However, this study still has certain limitations. The sample size of this study was not very large, which may have had an impact on the study. Further comprehensive studies with large samples sizes are needed.

## Data Availability

The datasets used or analysed during the current study are available from the corresponding author on reasonable request.
